# Lactate Dehydrogenase B Is Associated with the Response to Neoadjuvant Chemotherapy in Oral Squamous Cell Carcinoma

**DOI:** 10.1371/journal.pone.0125976

**Published:** 2015-05-14

**Authors:** Wenyi Sun, Xiaomin Zhang, Xu Ding, Huaiqi Li, Meiyu Geng, Zuoquan Xie, Heming Wu, Min Huang

**Affiliations:** 1 Division of Antitumor Pharmacology, State Key Laboratory of Drug Research, Shanghai Institute of Materia Medica, Chinese Academy of Sciences, Shanghai, China; 2 Jiangsu Key Laboratory of Oral Diseases, Department of Oral and Maxillofacial Surgery, Affiliated Hospital of Stomatology, Nanjing Medical University, Nanjing, China; Winship Cancer Institute of Emory University, UNITED STATES

## Abstract

Oral squamous cell carcinoma (OSCC) comprises a subset of head and neck squamous cell carcinoma (HNSCC) with poor therapeutic outcomes and high glycolytic dependency. Neoadjuvant chemotherapy regimens of docetaxel, cisplatin and 5-fluorouracil (TPF) are currently accepted as standard regimens for HNSCC patients with a high risk of distant metastatic spread. However, the antitumor outcomes of TPF neoadjuvant chemotherapy in HNSCC remain controversial. This study investigated the role of lactate dehydrogenase B (LDHB), a key glycolytic enzyme catalyzing the inter-conversion between pyruvate and lactate, in determining chemotherapy response and prognosis in OSCC patients. We discovered that a high protein level of LDHB in OSCC patients was associated with a poor response to TPF regimen chemotherapy as well as poor overall survival and disease-free survival. Our in-depth study revealed that high LDHB expression conferred resistance to taxol but not 5-fluorouracil or cisplatin. LDHB deletion sensitized OSCC cell lines to taxol, whereas the introduction of LDHB decreased sensitivity to taxol treatment. Taxol induced a pronounced impact on LDHB-down-regulated OSCC cells in terms of apoptosis, G2/M phase cell cycle arrest and energy metabolism. In conclusion, our study highlighted the critical role of LDHB in OSCC and proposed that LDHB could be used as a biomarker for the stratification of patients for TPF neoadjuvant chemotherapy and the determination of prognosis in OSCC patients.

## Introduction

Oral squamous cell carcinoma (OSCC) is a subset of head and neck squamous cell carcinoma (HNSCC) that has a poor therapeutic outcome and a 5-year survival rate of 50%–60% [1, 2]. Despite advances in multidisciplinary treatment modalities, no improvement in the 5-year survival rate has been achieved over the past 20 years [3]. Recently, neoadjuvant chemotherapy has emerged as an effective way to reduce locally advanced or aggressive cancers to improve the chance of eradicating locoregional lesions by radical surgery and/or radiation in HNSCC patients. Several randomized trials have discovered that a neoadjuvant chemotherapy regimen of docetaxel, cisplatin and 5-fluorouracil (TPF) improved overall survival and progression-free survival in HNSCC patients [4–7]. Indeed, TPF has been accepted as a standard regimen for HNSCC patients with a high risk of distant metastasis; nonetheless, it was noted that TPF failed to demonstrate a survival advantage in the overall study population [8]. In addition, a meta-analysis showed that neoadjuvant chemotherapy decreased the rate of distant metastasis but did not improve the survival of HNSCC patients [9]. These discrepancies raise the possibility that TPF neoadjuvant chemotherapy might improve antitumor outcomes in a molecularly defined subset of patients. As such, the identification of well-defined molecular signatures is essential for decreasing the risk of surgery delay in patients with chemotherapy-resistant tumors.

OSCC is known as a type of highly hypoxic cancer, which may suggest its unique metabolic profile of glycolytic dependency. In cancer cells with high aerobic glycolysis, glucose is preferentially converted into lactic acid, known as the "Warburg effect" [10], and lactate dehydrogenase (LDH) plays an essential role by catalyzing the formation of lactic acid from pyruvate. In fact, it has been shown that the overexpression of LDH conferred glycolytic dependency in some tumor subtypes. LDH is a tetrameric enzyme composed of two major subunits, A and/or B, resulting in five isozymes: A4 (LDH-5), A3B1 (LDH-4), A2B2 (LDH-3), A1B3 (LDH-2) and B4 (LDH-1). LDHA, the predominant subunit in skeletal muscle, catalyzes the conversion of pyruvate into lactate, whereas LDHB, mainly expresses in heart muscle, favors the conversion of lactate into pyruvate [11]. The role of LDHA in cancer malignancy has been intensively studied to date. LDHA is elevated and activated in many cancers [12–15] and is believed to play a crucial role in tumor initiation [16], maintenance and progression [17]. LDHA is transcriptionally regulated by the oncogenes c-MYC [18] and Hif-1 [19] or directly phosphorylated by the tyrosine kinase FGFR1 to promote aerobic glycolysis [20]. The inhibition of LDHA leads to oxidative stress and subsequent mitochondrion-dependent apoptosis in cancer cells [21, 22].

However, the significance of LDHB in tumor progression and therapeutic outcomes remains elusive and not well characterized. An increasing number of studies have shown that LDHB might play a critical role in some subtypes of cancers. LDHB expression was found to correlate with both KRAS genomic copy number gains and KRAS mutations in lung cancer, which might explain why KRAS-mutant lung tumors are highly dependent on glycolysis for proliferation [23]. In basal-like or triple-negative breast cancer, LDHB was highly expressed and associated with poor outcomes [24]. Furthermore, the level of LDHB predicted a pathologically complete response to anthracycline-based neoadjuvant chemotherapy in both HR-positive/HER2-negative and triple-negative breast cancers [25]. These findings suggest a critical role of LDHB in a subset of human cancers.

In this study, we investigated the role of LDHB in determining the response to chemotherapy in OSCC. We discovered that a high level of LDHB expression was correlated with a low response to TPF neoadjuvant chemotherapy, which was possibly due to compromised sensitivity to taxol treatment. High LDHB expression was also closely associated with poor overall survival and disease-free survival in OSCC. These observations supported LDHB as a predictive marker for the response to TPF neoadjuvant chemotherapy in patients with OSCC and as a prognostic marker for OSCC.

## Materials and Methods

### Patient samples

Human paraffin-embedded tissue samples were collected from 107 patients (61 males and 46 females) who were examined and treated for primary OSCC at the Stomatological Hospital of Nanjing Medical University, Nanjing, China, from Jan 2005 to Dec 2008. Written informed consent from these patients was obtained for the use of their tissue samples and for follow-up interviews for the research. The tumor grade was classified as poorly differentiated, moderately differentiated, and well differentiated, and the pathological stage was defined according to the American Joint Committee on Cancer (AJCC) TNM staging system. Primary tumor sites included the tongue (n = 40), gingiva (n = 22), buccal mucosa (n = 30), mouth floor (n = 3), palate (n = 7), and jaw (n = 5). Follow-up data were collected through direct interviews with the patients or their relatives. From Jan 2010 to Jun 2014, another set of 50 patients with locally advanced resectable OSCC (stage III or IVA) underwent two cycles of TPF induction chemotherapy (75 mg/m^2^ of docetaxel on day 1, 75 mg/m^2^ of cisplatin on day 1, and 750 mg/m^2^ of fluorouracil on day 1 to day 5), and the tumor response to induction chemotherapy was assessed according to RECIST (version 1.0) by standard evaluation and CT/MRI imaging studies prior to radical resection [8]. A favorable response was defined as a complete or partial response (sensitive); an unfavorable response was defined as a stable or progressive disease (resistant).

### Ethics Statement

The study was conducted in accordance with the guidelines of the Declaration of Helsinki. The ethical review board (Committee of Ethics of Nanjing Medical University) approved the use of human paraffin-embedded tissues.

### Immunohistochemistry

LDHB expression was determined by immunohistochemical staining using an LDHB antibody (Rabbit polyclonal, Cat No: ab75167, Abcam, MA, USA) diluted 1:100, according to the manufacturer’s instructions. Negative controls consisted of tissue sections were incubated with normal mouse serum or phosphate-buffered saline (PBS). All of the sections were counterstained with hematoxylin. LDHB expression was evaluated semi-quantitatively as the total immunostaining score, which was calculated as the product of the proportion score and the intensity score. Briefly, the proportion score was defined as the fraction of positively stained cells: 1, <5%; 2, 5–10%; 3, 10–50%; 4, 50–75%; 5, >75% of cell stained. The staining intensity was evaluated as follows: 0, no staining signal; 1, weak positive signal; 2, moderate positive signal; 3, strong positive signal. Thus, the total expression score could range from 0 to 15. High LDHB expression was defined as a total expression score ≥7. The evaluation was performed independently by two observers, and the average of two readings was used for the statistical analysis.

### Cell culture

Cal27 cells were obtained from American Type Culture Collection (MD, USA). Tca8113 and KB cells were bought from the China Center for Type Culture Collection (Hubei, China). HN4, HN6, HN12 and HN30 cells were provided by Shanghai Ninth Hospital of Shanghai Jiaotong University School of Medicine, generously donated by Dr. Silvio Gutkind on Dec. 2011 (NIH, MD, USA), and were used in previous studies [26–29]. The Cal27 and HN series cells were maintained in DMEM, KB cells in MEM and TCA8113 cells in RPMI-1640 supplemented with 10% fetal bovine serum (FBS) in a humidified incubator containing 5% CO_2_ at 37°C. All of the culture media and FBS were purchased from Invitrogen (CA, USA).

### siRNA transfection

siRNA oligonucleotides for LDHA and LDHB were synthesized by Raybiotech (Guangdong, China), with a scrambled siRNA used as a control. Transfection was performed using Lipofectamine RNAiMAX (Life Technologies, IL, USA) according to the manufacturer’s protocol. Whole-cell lysates were prepared for further analysis at 72 h post-transfection. The target sequences for LDHB were 5-CUGGAAACUAAGUGGAUUATT-3 and 5-CCCGUGUCAACAAUGGUAATT-3, and the target sequences for LDHA were 5-GGAGAAAGCCGUCUUAAUU-3 and 5-GGCAAAGACUAUAAUGUAA-3.

### Western blot analysis

Cells were lysed with RIPA Buffer (25 mM Tris-HCl (pH 7.6), 150 mM NaCl, 1% NP-40 (v/v), 1% sodium deoxycholate (w/v), 0.1% SDS (w/v) and protease inhibitor cocktail), and the amount of total protein was determined with a BCA protein assay kit (Beyotime, Jiangsu, China). Equal amounts of protein (10 μg) were separated by electrophoresis on a 10% (w/v) PAGE gel and transferred to an Immun-Blot PVDF membrane (Bio-Rad, CA, USA). The membranes were blocked in 3% (w/v) BSA in Tris-buffered saline/Tween 20 buffer and then immunoblotted with the corresponding antibodies (diluted in 1:1000) obtained as follows: LDHA (Rabbit polyclonal, Cat No: ab47010), LDHB (Rabbit polyclonal, Cat No: ab75167), and GAPDH (Rabbit monoclonal, Cat No: ab181602) from Abcam (MA, USA), bcl-2 (Rabbit monoclonal, Cat No: #4223S), caspase-3 (Rabbit monoclonal, Cat No: #9665), and caspase-7 (Rabbit monoclonal, Cat No: #12827) from CST (MA, USA), cytochrome C (Mouse monoclonal, Cat No: AC909) from Beyotime (Jiangsu, China). The primary antibodies were visualized with HRP-conjugated secondary antibodies using Western Lightning-ECL (Millipore, MA, USA).

### ATP assay

Intracellular ATP levels were assessed using an ATP assay kit (BioVision, CA, USA). Cells were lysed in 200 μL of lysis buffer and centrifuged at 12,000 ×*g* to collect the supernatant. An aliquot (100 μL) of ATP detection working solution was added to each well of a black 96-well culture plate (Corning, MA, USA), and the plate was incubated for 5 min at room temperature. Then, 10 μL of the cell lysate was added to the wells, and the luminescence was measured immediately. The data were normalized to the amount of protein content in each sample.

### Oxygen consumption rate (OCR) and extracellular acidification rate (ECAR) analyses

The XF96 extracellular flux analyzer (Seahorse Biosciences, MA, USA) was used to measure oxygen consumption, expressed in pmol/min, and the extracellular acidification rate, expressed in mpH/min. Cells were seeded in XF96 cell culture plates at a density of 8000 cells/well. After attachment, compounds were added and incubated for 24 h. The cells were equilibrated with bicarbonate-free buffered DMEM at 37°C for 1 h without CO_2_ immediately before the XF assay. Each XF96 assay well was equipped with a disposable sensor cartridge and embedded with 96 pairs of fluorescent biosensors (oxygen and pH) coupled to fiber-optic waveguides that delivered light rays at various excitation wavelengths (oxygen = 532 nm, pH = 470 nm) and transmitted a fluorescent signal (oxygen = 650 nm, pH = 530 nm) to a set of highly sensitive photodetectors.

### Cell cycle analysis

Cell cycle distributions were determined by propidium iodide (PI, Sigma-Aldrich, MO, USA) staining. In brief, 2×10^5^ of cells in a 6-well culture dish were incubated with the indicated doses of taxol for 72 h. The cells were then washed twice with PBS and fixed in 70% (v/v) ethanol for 1 h. The cells were washed again with PBS and then incubated with PI (10 mg/mL) and RNase A at 37°C for 30 min. The cell cycle distributions were measured with a Flow Cytometer (Becton Dickinson, CA, USA) and analyzed using FlowJo software.

### Apoptosis assay

Apoptosis was measured by annexin V and PI staining using the Annexin V-FITC Apoptosis Detection Kit (Vazyme, Jiangsu, China), according to the manufacturer’s protocol, and analyzed by flow cytometry.

### Cell viability assay

Cells were seeded at a density of 3×10^3^ per well in 100 μL of culture medium in a 96-well plate. Shortly after attachment, the cells were treated with taxol at the indicated dose. After incubation for 72 h, a CCK8 assay was performed according to the manufacturer’s protocol (Life Technologies, IL, USA).

### Mitochondrion-free cytoplasm preparation

Mitochondria were isolated from KB cells using Cell Mitochondria Isolation Kit (Beyotime, Jiangsu, China). Briefly, cells were collected, washed with PBS, and then suspended in ice-cold isolation buffer for 15 min. After the cells were homogenized, the homogenate was centrifuged at 600×*g* for 10 min at 4°C, and the supernatant was then centrifuged at 11,000×*g* for 10 min at 4°C. The mitochondria were collected in the pellet, and the supernatant was considered the cytosol without mitochondria.

### Mitochondrial cytochrome C assay

We used Millipore’s FlowCellect Cytochrome C Kit (MA, USA) to assess the loss of mitochondrial cytochrome C in cells. Briefly, mitochondria were isolated and detected using an anti-Cytochrome C-FITC antibody and then analyzed and sorted by FACS analysis.

### Clonogenic Assay

Cells were seeded at a density of 1×10^2^ in a 6-well plate and treated with taxol at the indicated dosage. After 10 days of culture, the colonies were fixed with ethanol and stained with 0.05% crystal violet (Sigma-Aldrich, MO, USA).

### LDH activity assay

The LDH activity of cell lysates was examined using Lactate Dehydrogenase Activity Assay Kit according to the manufacturer’s instructions (BioVision, CA, USA). In brief, cells were collected, washed and extracted to measure LDH activity. The results were normalized to the amount of total protein.

### Extracellular lactate measurement

Cells were cultured in serum-free medium and their extracellular lactate level was measured using a lactate assay kit (Beyotime, Jiangsu, China). The readouts were normalized by the corresponding protein amounts.

### Statistical analysis

Comparisons between groups were analyzed by a one-way ANOVA using statistical package SPSS (version 19.0; SPSS Inc., IL, USA), and *P* values less than 0.05 were considered statistically significant. The statistical significance of the difference between LDHB expression and TPF-induced chemotherapeutic efficacy was examined using cross-table and Chi-square analyses. The statistical significance of the difference between LDHB expression and pathological parameters was examined using a Chi-square analysis. Overall survival and disease-free survival were calculated by the Kaplan-Meier method and compared with the log-rank test.

## Results

### LDHB is associated with OSCC prognosis and response to chemotherapy

**Fig 1:**
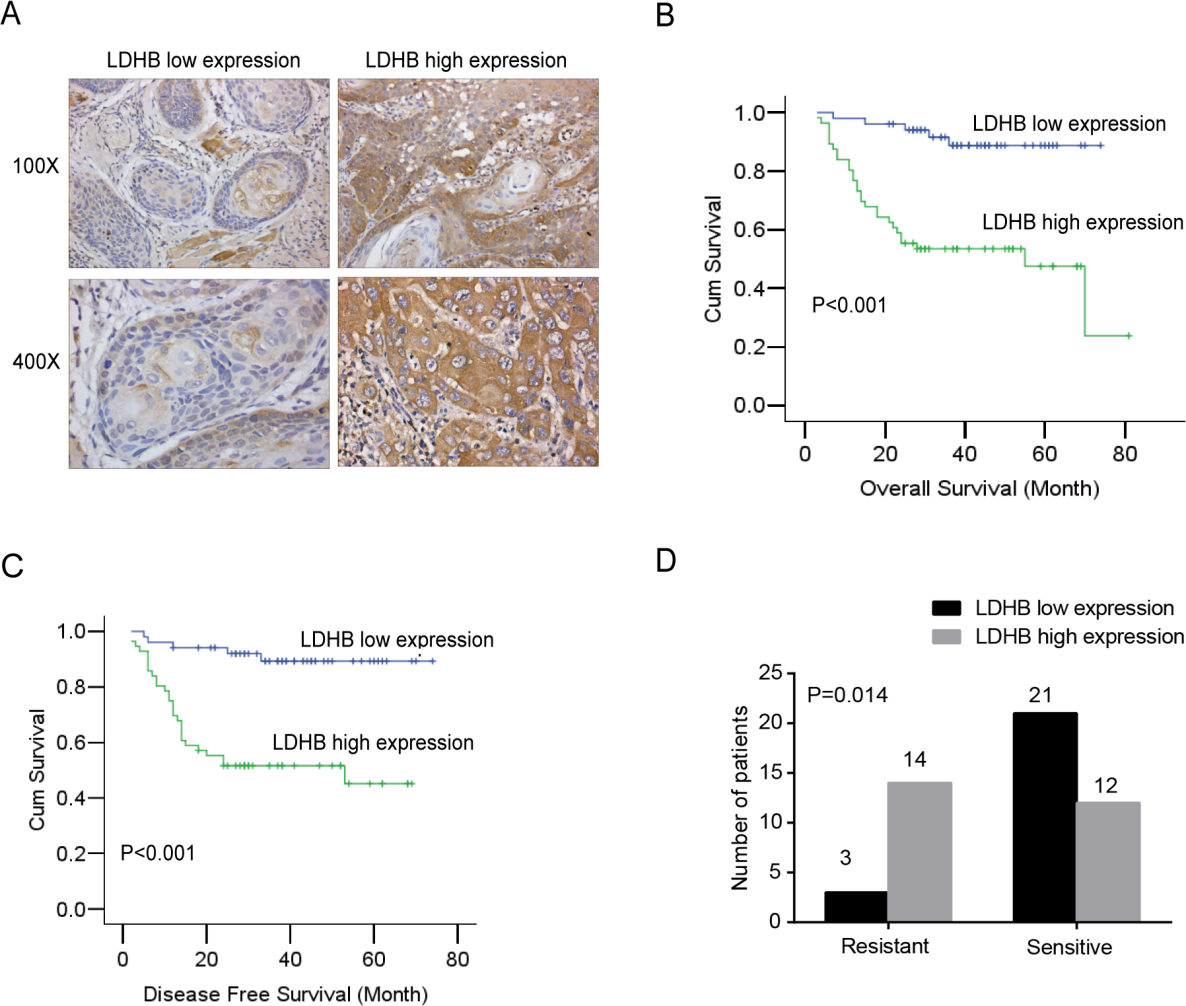
LDHB is associated with prognosis and TPF induction chemotherapy in OSCC patients. (A) Representative images of immunohistochemical staining for LDHB expression in OSCC patients. (B, C) The correlation of LDHB expression with overall survival and disease-free survival in OSCC patients (n = 107), as determined by Kaplan-Meier survival analysis. The *P* value was calculated using the log-rank test. (D) The correlation between LDHB expression and TPF induction chemotherapy efficacy in OSCC patients, as determined by cross-table analysis (n = 50).

First, we examined the expression of LDHB in a total of 107 OSCC patient samples using immunohistochemical staining. The patients were then classified into LDHB low-expression and high-expression groups according to the proportion and intensity scores (Fig 1A). We observed that a high expression of LDHB was significantly associated with poor overall survival (*P <* 0.001) and disease-free survival (*P <* 0.001) (Fig 1B and 1C). Moreover, we compared the expression of LDHB with pathological parameters (Table 1) and found that LDHB expression was correlated with tumor recurrence (*P* = 0.011) and closely correlated with tumor size (*P* = 0.090), distant metastasis (*P* = 0.055) and TNM classification (*P* = 0.079) but was not significantly correlated with sex, age, histological grade or lymphatic metastasis. As LDHB is closely associated with a poor prognosis, to further examine its medical application, we analyzed the correlation between LDHB and the response to TPF neoadjuvant chemotherapy in a new set of OSCC patients. Intriguingly, we found a significant correlation between LDHB expression and the response to TPF neoadjuvant chemotherapy in OSCC patients (*P* = 0.014) (Fig 1D). A high LDHB expression was correlated with an unfavorable response to TPF neoadjuvant chemotherapy, and the majority of patients in this group were resistant to therapy (14/26). Conversely, patients with low LDHB expression were more responsive to TPF neoadjuvant chemotherapy (21/24).

**Table 1:** Correlations between LDHB and pathological parameters.

Parameter	Low LDHB	High LDHB	*P* value
Age			1.0
< 60 years	25	28	
≥ 60 years	26	28	
Sex			0.441
Female	24	22	
Male	27	34	
Tumor size			0.090*
< 2 cm^3^	7	8	
≥ 2 cm^3^, < 4 cm^3^	36	28	
≥ 4 cm^3^	8	20	
TNM classification			0.079*
I or II	29	23	
III or IV	22	33	
Regional metastasis			0.329
With	19	27	
Without	32	29	
Distant metastasis			0.055
With	2	9	
Without	49	47	
Recurrence			0.011*
With	6	19	
Without	45	37	
Histological grading			0.285
Grade I	32	31	
Grade II	17	20	
Grade III	2	5	
OSCC location			0.464
Tongue	23	18	
Buccal	14	15	
Gingiva	6	14	
Others (hard palate, mouth floor, etc)	8	9	

*Statistically significant difference.

### The level of LDHB is correlated with OSCC cell sensitivity to taxol treatment

**Fig 2:**
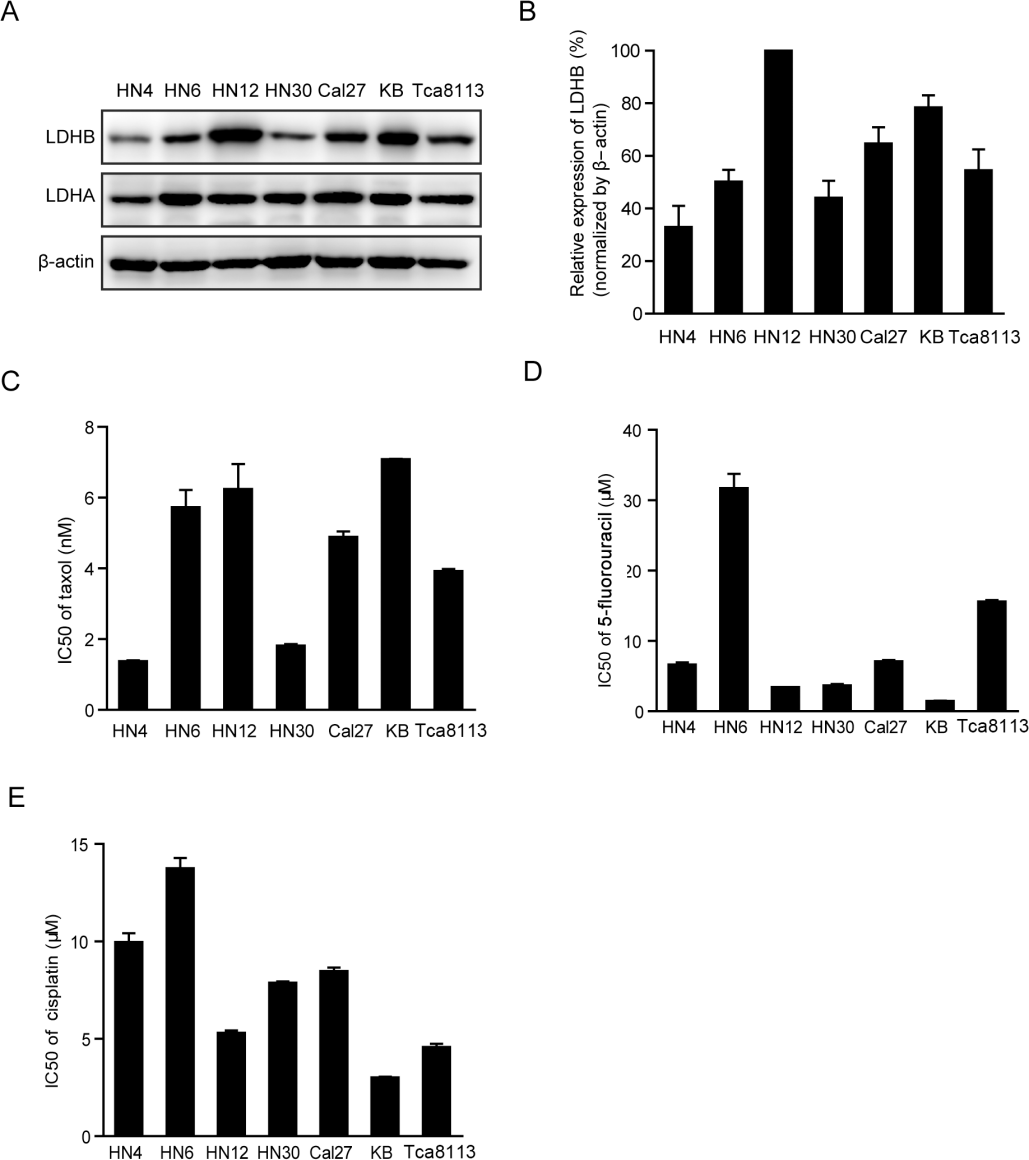
Correlation between LDHB expression and the cell-inhibitory potency of taxol. (A) A western blot analysis of LDHA and LDHB in seven OSCC cell lines. (B) The quantification of LDHB relative expression in three independent experiments. (C) The IC_50_ value of taxol in seven OSCC cell lines. (D) The IC_50_ value of 5-fluorouracil in seven OSCC cell lines. (E) The IC_50_ value of cisplatin in seven OSCC cell lines. Mean ± SE (n = 3).

We examined the protein levels of LDHA and LDHB in seven OSCC cell lines. As shown in Fig 2A and 2B, the protein level of LDHB varied among different OSCC cell lines, whereas the LDHA level was very similar. We then probed the role of LDHB in OSCC progression by examining the impact of LDHB knockdown on the proliferation of these cell lines. Results showed that the growth inhibition varied in the range of 3.32% to 39.42% (S1 Fig), suggesting that the effect of LDHB knockdown on tumor progression might be dependent on its basal level in tumors. Next, we examined whether the LDHB level was correlated with the sensitivity to each component of TPF regimens, namely, taxol, cisplatin and 5-fluorouracil. Interestingly, we found that the level of LDHB in these cell lines was closely correlated with the efficacy of taxol (Fig 2C) but not 5-fluorouracil or cisplatin (Fig 2D and 2E). KB and HN12 cells, which possessed the highest abundance of LDHB, displayed the highest IC_50_ values (7.09 nM and 6.24 nM, respectively), whereas HN4 and HN30, with the lowest expression of LDHB, displayed the highest sensitivity to taxol, with IC_50_ values of 1.39 nM and 1.82 nM, respectively.

### LDHB affects the sensitivity of OSCC cells to taxol

**Fig 3:**
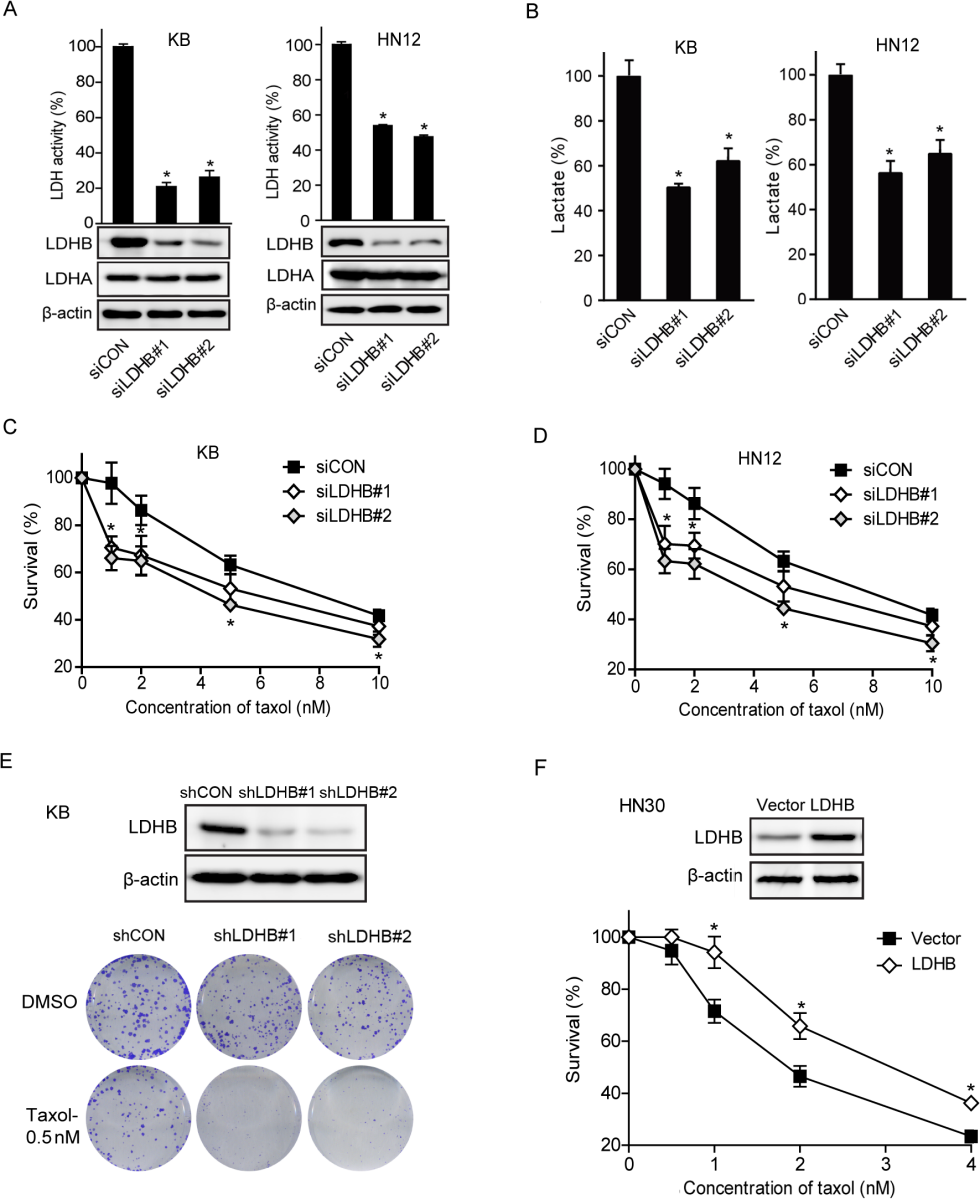
LDHB impacts the efficacy of taxol. (A, B) KB and HN12 cells were transfected with scrambled or LDHB siRNA. (A) After 72 h, LDHB expression was examined, and LDH activity was measured. (B) After 48 h, cells were washed with PBS twice followed by the addition of fresh serum-free media and extracellular lactate amount was measured at 12 h post-incubation. (C, D) KB and HN12 cells were transfected with scrambled or LDHB siRNA and then treated with DMSO or taxol for 72 h at the indicated concentrations, and cell viability was measured. (E) The LDHB stable-interference strains of KB cells were established and verified by western blotting; the clonogenic assay was conducted in shCON and shLDHB KB cells exposed to 0.5 nM taxol. (F) HN30 cells were transfected with empty vector or LDHB and treated with DMSO or taxol for 72 h at the indicated concentrations. *P < 0.05, compared to the control group. Mean ± SE (n = 3).

The above results suggested that the level of LDHB might be a determinant of cell sensitivity to taxol. Therefore, we knocked down the expression of LDHB in KB and HN12 cells using siRNA. In consistent with the decrease of LDH activity, lactate level was markedly reduced by LDHB knockdown (Fig 3A and 3B), indicating that LDHB was the critical subtype of LDH that catalyzed the conversion of pyruvate into lactate in these cells. In parallel, LDHB depletion sensitized both KB and HN12 cells to taxol treatment (Fig 3C and 3D). Cell viability after taxol treatment was remarkably decreased in LDHB-depleted cells compared to the control cells. This observation was recapitulated in a clonogenic assay (Fig 3E). In contrast, the knockdown of LDHB did not affect the potency of either 5-fluorouracil or cisplatin in KB cells (S2 Fig). Meanwhile, no changes in LDH activity or lactate production were detected in both cells following taxol treatment for 12 h, ruling out the possibility of LDHB as a direct target of taxol (S3 Fig). Consistently, LDHB overexpression in HN30 cells, which have low level of endogenous LDHB, decreased the sensitivity of these cells to taxol (Fig 3F). We also examined the impact of LDHA on cell sensitivity to taxol treatment and found that LDHA depletion did not show any effect on the sensitivity of either KB or HN12 cells to taxol (S4 Fig).

### LDHB depletion enhances taxol-induced G2/M arrest and apoptosis

**Fig 4:**
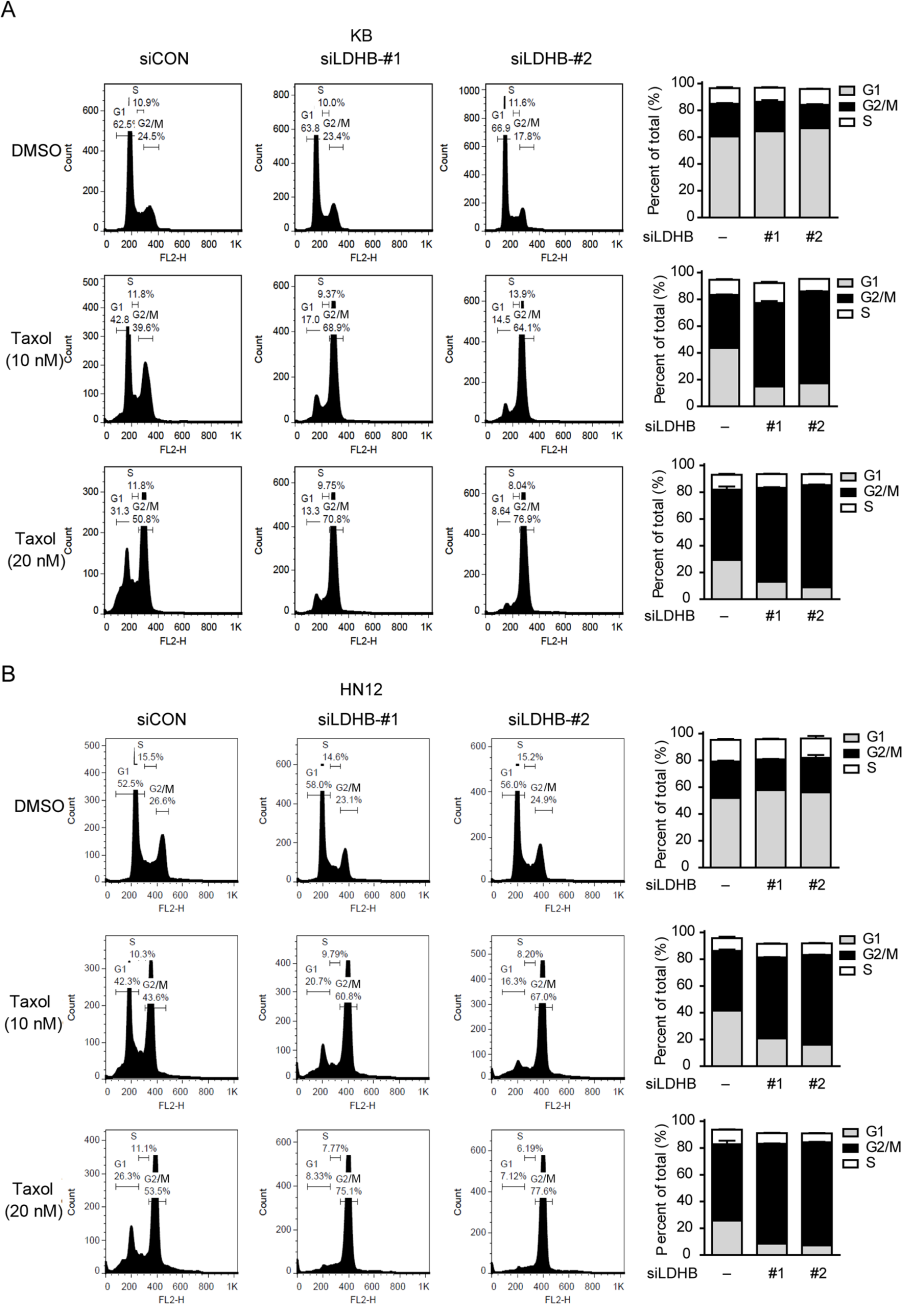
Silencing LDHB enhances taxol-induced G2/M arrest. (A, B) KB and HN12 cells were transfected with scrambled or LDHB siRNA and treated with DMSO or taxol for 12 h at the indicated concentrations. The cell cycle was measured by flow cytometry with PI staining. Quantification results from three independent experiments are shown on the right side.

Taxol is an anti-microtubule agent that inhibits cell growth by arresting cancer cells in mitosis and inducing subsequent apoptosis. To understand how LDHB influences the anticancer effect of taxol, we examined the effect of LDHB on cell cycle progression. LDHB siRNA per se did not affect the cell cycle distribution, whereas the knockdown of LDHB exaggerated taxol-induced G2/M phase arrest in KB and HN12 cells (Fig 4). Taxol (10 nM) alone led to only 15% G2/M phase arrest, whereas the proportion increased to over 40% in LDHB-silenced cells.

**Fig 5:**
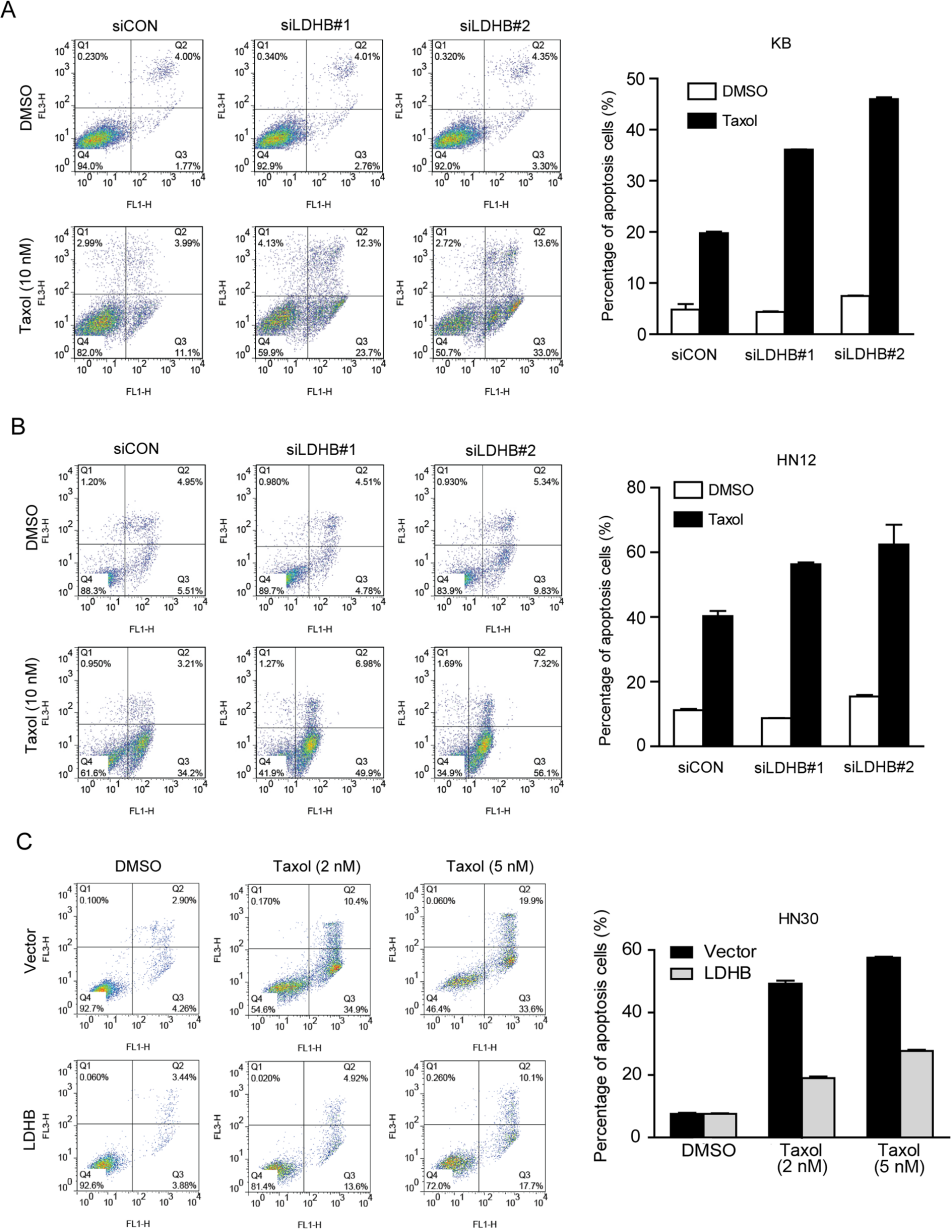
Knockdown of LDHB enhances taxol-induced apoptosis. (**A, B**) KB cells and HN12 cells were transfected with scrambled or LDHB siRNA and treated with DMSO or 10 nM taxol for 48 h. **(C)** HN30 cells were transfected with empty vector or LDHB and treated with DMSO or taxol for 48 h at the indicated concentrations. Quantification results of three independent experiments are shown on the right side.

**Fig 6:**
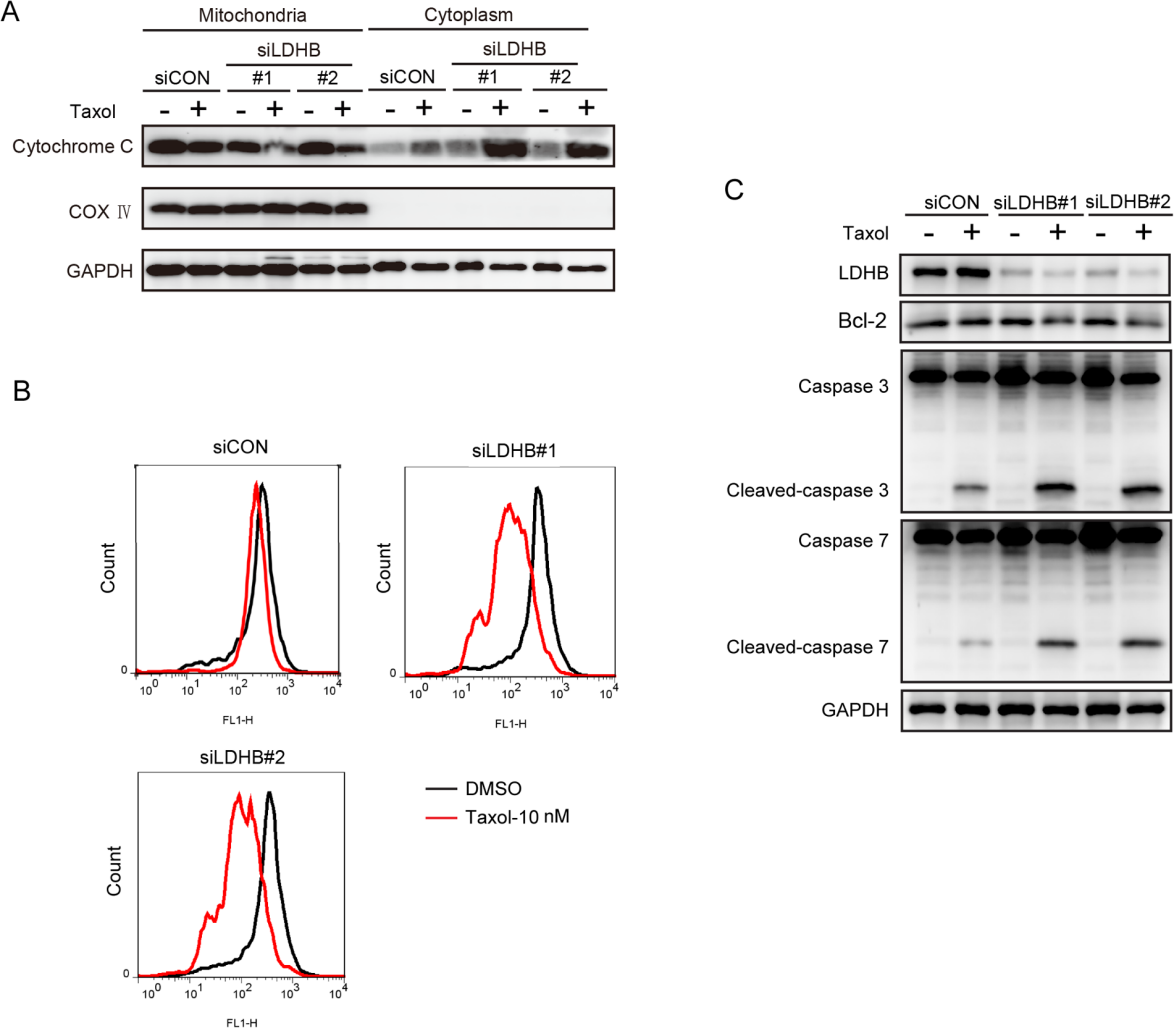
Knockdown of LDHB induces the mitochondrion-dependent apoptosis pathway. KB cells were transfected with scrambled or LDHB siRNA and then treated with DMSO or 10 nM taxol for 24 h. (**A**) The mitochondria and the cytoplasm were separated and measured for cytochrome C by western blotting. COX IV was used as a marker for mitochondria. (**B**) Mitochondrial cytochrome C was measured by FACS analysis. (**C**) Cell lysates were prepared for western blotting. GAPDH was used as a loading control.

We also measured taxol-induced apoptosis. In agreement with the cell-cycle effect, the knockdown of LDHB did not induce apoptosis in either KB or HN12 cells but dramatically enhanced the taxol-induced apoptotic effect (Fig 5A and 5B). These observations were further supported by the promoted molecular events implicated in apoptosis. Compared to the control cells, the accumulation of cytoplasm cytochrome C was dramatically increased by taxol treatment in LDHB knockdown cells, in line with the reduction of mitochondrial cytochrome C (Fig 6A and 6B). We also observed higher levels of cleaved caspase 3 and caspase 7 and reduced expression of bcl-2 in LDHB knockdown KB cells after exposure to taxol for 24 h (Fig 6C). Consistently, the overexpression of LDHB remarkably reversed the apoptotic effect induced by taxol in HN30 cells (Fig 5C), further supporting that LDHB is a determinant of the cellular impact of taxol.

### LDHB strengthens the effect of taxol on glucose metabolism

OSCC is known as a type of highly hypoxic cancer. It has been extensively reported that hypoxia could desensitize cancer cells to chemotherapeutic drugs [30, 31]. It was thus reasonable to speculate that LDHB might have a role in hypoxia, thus affecting the sensitivity to taxol. However, the expression of HIF-1α, a well-accepted molecular indicator of hypoxic status [32], seemed unaffected by LDHB interference (S5 Fig), largely excluding the possibility that LDHB modulated the cell sensitivity to taxol via its impact on hypoxia.

**Fig 7:**
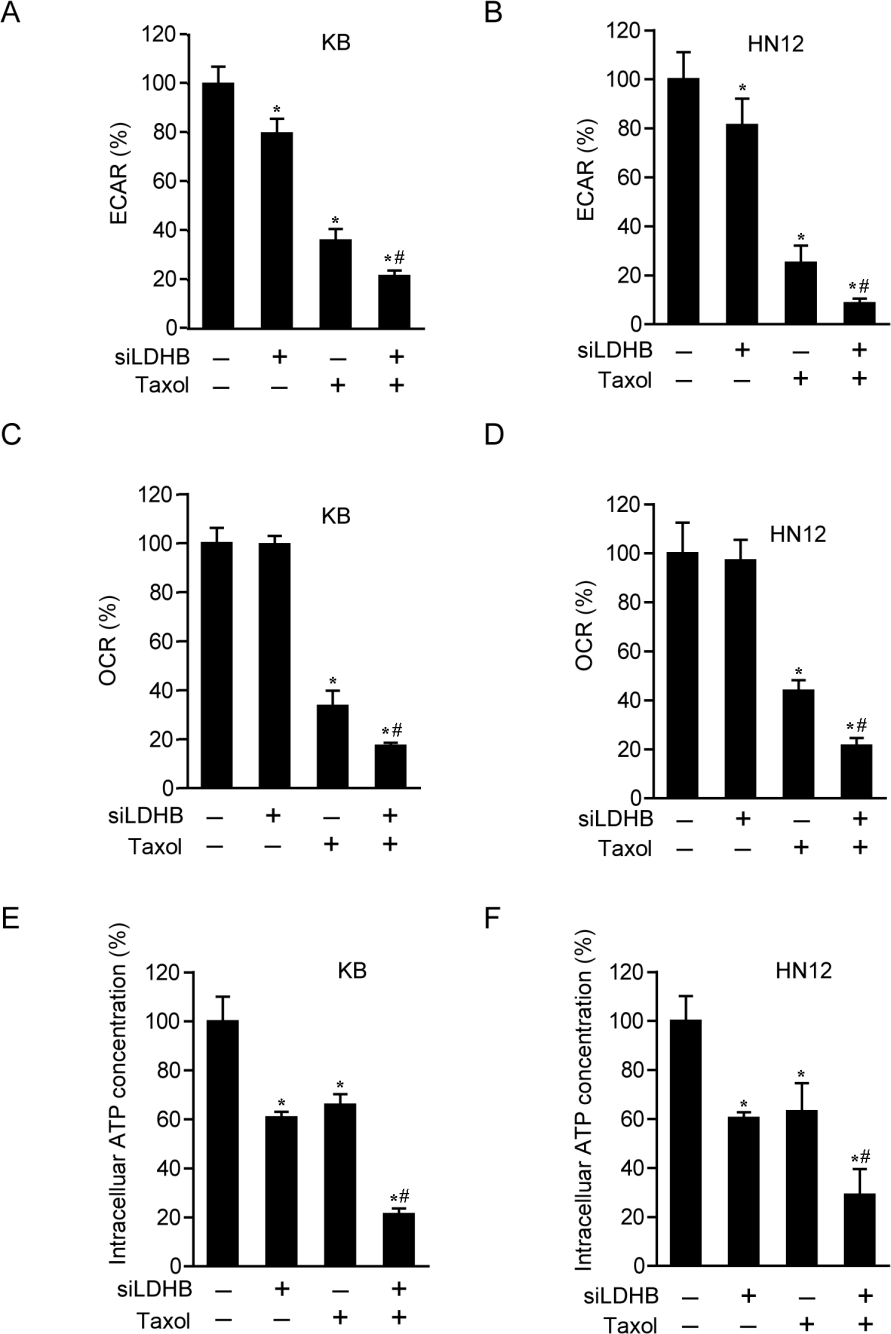
Combination of taxol and LDHB down-regulation exhibits a synergistic effect on cell metabolism. KB and HN12 cells were transfected with scramble or LDHB siRNA and then treated with DMSO or taxol of 10 nM for 24 h. (**A, B**) ECAR was measured by an XF96 analyzer. (**C, D**) OCR was measured by an XF96 analyzer. (**E, F**) Cell lysates were prepared, and the intracellular ATP level was determined. *P < 0.05, compared to the control group; ^#^ *P* < 0.05, compared to the taxol group. Mean ± SE (n = 3).

The highly hypoxic feature of OSCC may also suggest its unique metabolic profile of glycolytic dependency. This possibility is strengthened by the fact that LDHB is a rate-limiting enzyme of aerobic glycolysis critically catalyzing the conversion of pyruvate into lactate. We then addressed whether metabolic alterations could contribute to the improved therapeutic efficacy of taxol in LDHB-depleted cells. As such, the metabolic alterations were measured by a Seahorse XF 96 Analyzer. ECAR and OCR were measured to indicate the glycolysis rate and the mitochondrial respiration rate respectively. As expected, LDHB depletion significantly decreased ECAR, indicating the reduced glycolysis and consequent lactate production. In the meanwhile, lactate addition failed to compensate the pronounced sensitivity towards taxol caused by LDHB knockdown in both KB and HN12 cells. These results excluded the possibility that lactate per se was involved in the cytotoxicity triggered by taxol (S6 Fig), pointing to a broad impact resulted from glycolysis defects. Indeed, 24-h taxol treatment of LDHB-down-regulated cells remarkably diminished glycolysis and mitochondrial respiration compared with the control cells (Fig 7A–7D). These data were supported by the measurement of ATP production, an outcome of glucose metabolism. The amount of ATP was more significantly reduced by taxol in LDHB-down-regulated cells (Fig 7E and 7F).

These results suggested that metabolic alterations might be involved in the synergistic effect of taxol and LDHB depletion in OSCC cells.

## Discussion

TPF neoadjuvant chemotherapy has exhibited benefits for OSCC patients with a high risk of distant metastasis [4, 5]. However, some studies have shown a limited response in OSCC patients [9]. These controversial reports highlight the importance of discovering a biomarker to define the appropriate subtype of OSCC for TPF neoadjuvant chemotherapy. In this study, we identified LDHB as a metabolic marker for the response to TPF neoadjuvant chemotherapy: patients with high LDHB expression are likely to be resistant to chemotherapy, suggesting that TPF neoadjuvant chemotherapy is suitable for patients with low LDHB expression. Moreover, high expression of LDHB correlated with a remarkable reduction in overall survival and disease-free survival, whereas low expression of LDHB correlated with favorable overall survival and disease-free survival over 6 years of follow-up. The lack of response to chemotherapy might partially contribute to the observed compromised overall survival and disease-free survival. This study highlights LDHB as a biomarker for neoadjuvant chemotherapy and prognosis in OSCC patients, which is of great significance for future medical applications.

We also attempted to investigate how LDHB influenced the response to TPF neoadjuvant chemotherapy among OSCC patients. By comparing the expression of LDHB and LDHA in a panel of OSCC cell lines, we observed that LDHB expression varied among these cell lines, though LDHA displayed similar expression levels. Intriguingly, the expression of LDHB was associated with sensitivity to taxol but not cisplatin or 5-fluorouracil, indicating that variable sensitivity to taxol might account for the observed differential response to TPF treatment. To explore this possibility, we depleted LDHB in KB and HN12 cells, which have a high level of basal expression, and observed that the down-regulation of LDHB but not LDHA enhanced sensitivity to taxol. Consistently, the introduction of LDHB decreased sensitivity to taxol. It has previously been reported that the expression and activity of LDHA were increased in taxol-resistant MDA-MB-435 breast cancer cell lines and that the down-regulation of LDHA significantly increased sensitivity to taxol [9]. These results suggested that the role of LDHA and LDHB in conferring drug resistance to chemotherapy possibly varied among cell contexts.

Interestingly, LDHB is generally believed to favor the conversion of lactate into pyruvate, while LDHA preferentially converts pyruvate into lactate. However, this does not always appear to be the case. LDHB is associated with glycolytic phenotypes in triple-negative breast cancer [24, 25] and KRAS-dependent lung adenocarcinomas [23], in which LDHB is the predominant form for glycolysis. Our results also suggested that LDHB was the predominant form in OSCC, as the knockdown of LDHB greatly reduced the conversion of pyruvate into lactate in both KB and HN12 cells. Nonetheless, it remains unclear how LDHB inhibition causes a dramatic decrease in the conversion of pyruvate to lactate, which raises an important question about the biochemical activity of the LDHB isoform. Because LDHB and LDHA are known to form tetramers and affect each other, it is possible that the down-regulation of LDHB somehow affects LDHA activity and the forward reaction. An alternative explanation is that the LDHB function opposes that of LDHA or directs LDHA function within the context of OSCC [25]. Our data extend these initial observations and implicate LDHB as a metabolic regulator in OSCC. Accordingly, it would be of interest to determine the upstream pathways that regulate LDHB expression in OSCC.

Another intriguing finding is that LDHB specifically affected sensitivity of cancer cells to taxol but not to either cisplatin or 5-fluorouracil. Taxol is well known for targeting microtubules by stabilizing their structure, which is vital for mitotic activity and cellular motility. Taxol is also a potent inhibitor of chromosomal replication by blocking cells in G2/M phase [33]. There are several mechanisms involved in taxol resistance, including high expression of membrane P-glycoprotein, drug efflux pumps, the alteration of microtubule structure, drug-binding affinity and cell cycle deregulation [34–38]. Our results indicated that LDHB was a relevant metabolic marker contributing to taxol resistance. How is glycolysis mechanistically involved in the modulation of cell sensitivity to taxol remains unclear. One possible assumption is that impaired lactate production might account for the increased sensitivity to taxol. However, the addition of lactate could not compensate the proliferative suppression elicited by taxol in LDHB knockdown cells, largely ruling out this possibility. It appeared to us that in OSCC cells with high LDHB expression, the metabolic suppression caused by LDHB depletion endowed cells with susceptibility to stresses, like taxol treatment. For example, one of contributors could be the reduction in ATP levels, which not only affected the biological status of cancer cells but also impeded the efflux of taxol, leading to the intracellular accumulation of taxol.

## Conclusions

In summary, we observed that LDHB expression was associated with poor overall survival and disease-free survival in patients with OSCC. Importantly, high LDHB expression was correlated with a reduced response to TPF neoadjuvant chemotherapy. LDHB but not LDHA was related to taxol sensitivity, demonstrating that the former played a predominant role in the cellular sensitivity to this drug. Furthermore, the combination of LDHB down-regulation and taxol induced pronounced effects on cell apoptosis, G2/M phase arrest and energy metabolism. This study highlighted the critical role of LDHB in OSCC and proposes that LDHB could be used as a biomarker for the selection of patients for TPF neoadjuvant chemotherapy and the prediction of OSCC prognosis.

## Supporting Information

S1 Fig The impact of LDHB knockdown on the cell proliferation varies among seven OSCC cell lines. Seven OSCC cells were transfected with scrambled or LDHB siRNA and cell viability was measured at 24, 48 and 72 h post-transfection, respectively. The interfering efficiency of LDHB was measured by western blotting. Mean ± SE (n = 3).
(TIF)

S2 Fig Knockdown of LDHB has no influence on the antitumor effect of cisplatin and 5-fluorouracil. KB cells were transfected with scrambled or LDHB siRNA and then treated with DMSO or cisplatin/5-fluorouracil for 72 h at the indicated concentrations. Cell survival was examined by SRB. Mean ± SE (n = 3).
(TIF)

S3 Fig Taxol treatment for 12 h does not affect the LDH activity or lactate production. KB and HN12 cells were treated with taxol at the indicated concentrations. After 12 h, LDH activity and lactate amount were measured. Mean ± SE (n = 3).
(TIF)

S4 Fig Knockdown of LDHA has no influence on the antitumor effect of taxol. KB and HN12 cells were transfected with scrambled or LDHA siRNA and then treated with DMSO or taxol for 72 h at the indicated concentrations. Cell survival was examined by CCK8. Mean ± SE (n = 3).
(TIF)

S5 Fig LDHB knockdown did not affect the level of HIF-1α. The efficiency of LDHB knockdown was verified by western blotting, followed by the detection of the expression of Hif-1α.
(TIF)

S6 Fig The addition of lactate to LDHB knockdown cell culture medium could not compensate the effect of LDHB knockdown. KB and HN12 cells were transfected with scrambled or LDHB siRNA and cell sensitivity was measured in the presence or absence of 2 mM lactate. Cell viability was measured using CCK8 assay following 72-h exposure to vehicle or taxol treatment. Mean ± SE (n = 3).
(TIF)

S7 Fig Original unadjusted blots. (TIF)
