# Supplementary figures and images for: Lactate Dehydrogenase B Is Associated with the Response to Neoadjuvant Chemotherapy in Oral Squamous Cell Carcinoma
Source: PLoS One. 2015 May 14;10(5):e0125976. doi: 10.1371/journal.pone.0125976 (PMC4431727; doi:10.1371/journal.pone.0125976)

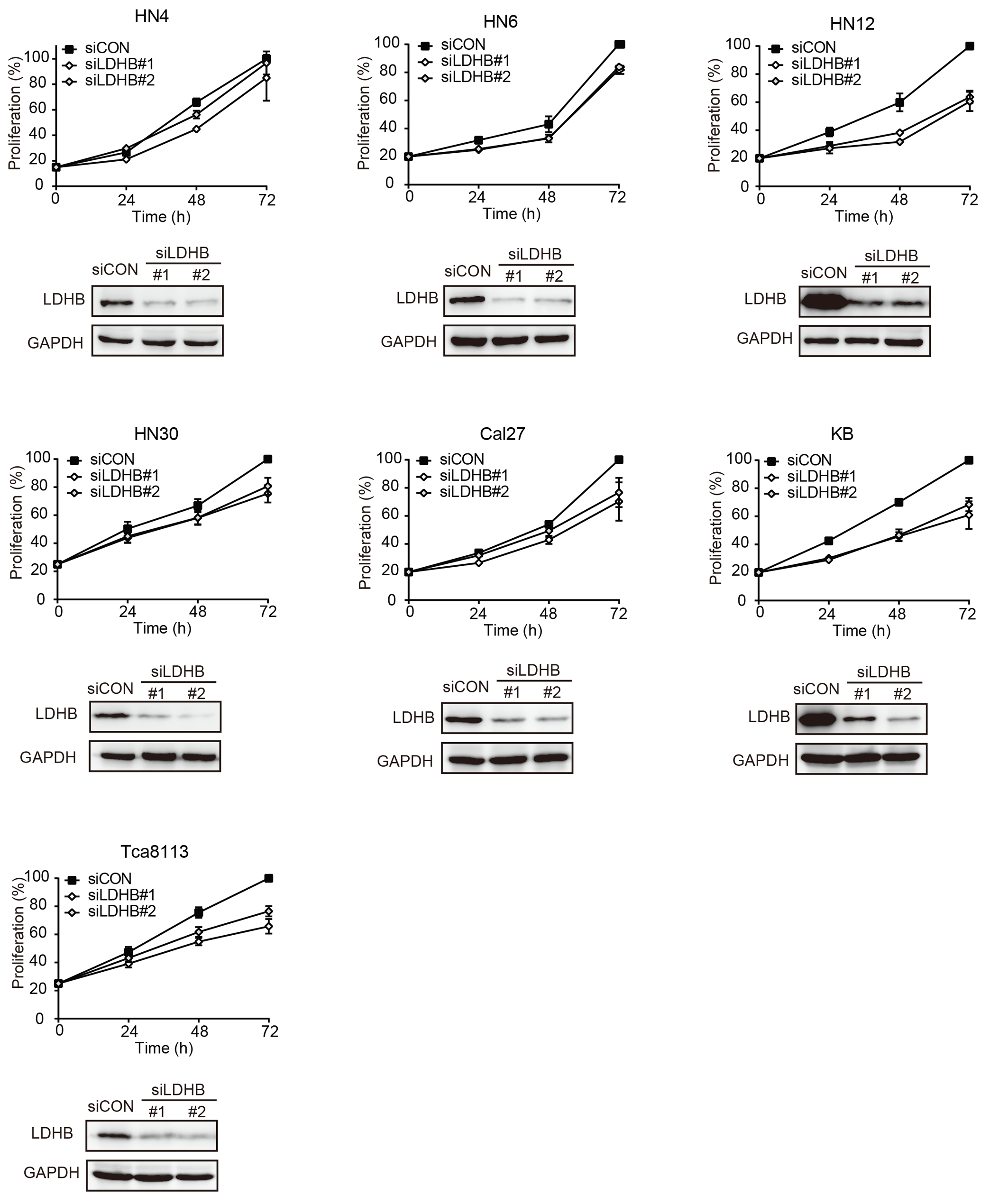

Supplement: S1 Fig — Seven OSCC cells were transfected with scrambled or LDHB siRNA and cell viability was measured at 24, 48 and 72 h post-transfection, respectively. The interfering efficiency of LDHB was measured by western blotting. Mean ± SE (n = 3). (TIF) [file pone.0125976.s001.tif]

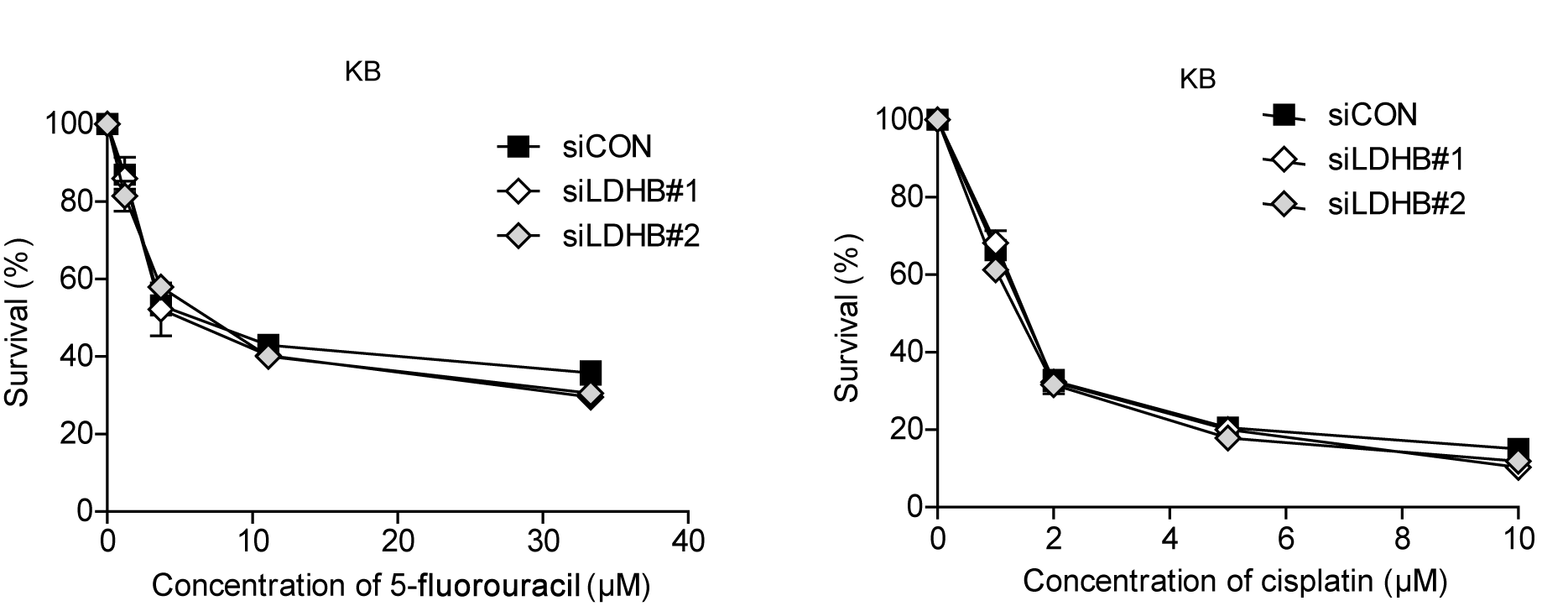

Supplement: S2 Fig — KB cells were transfected with scrambled or LDHB siRNA and then treated with DMSO or cisplatin/5-fluorouracil for 72 h at the indicated concentrations. Cell survival was examined by SRB. Mean ± SE (n = 3). (TIF) [file pone.0125976.s002.tif]

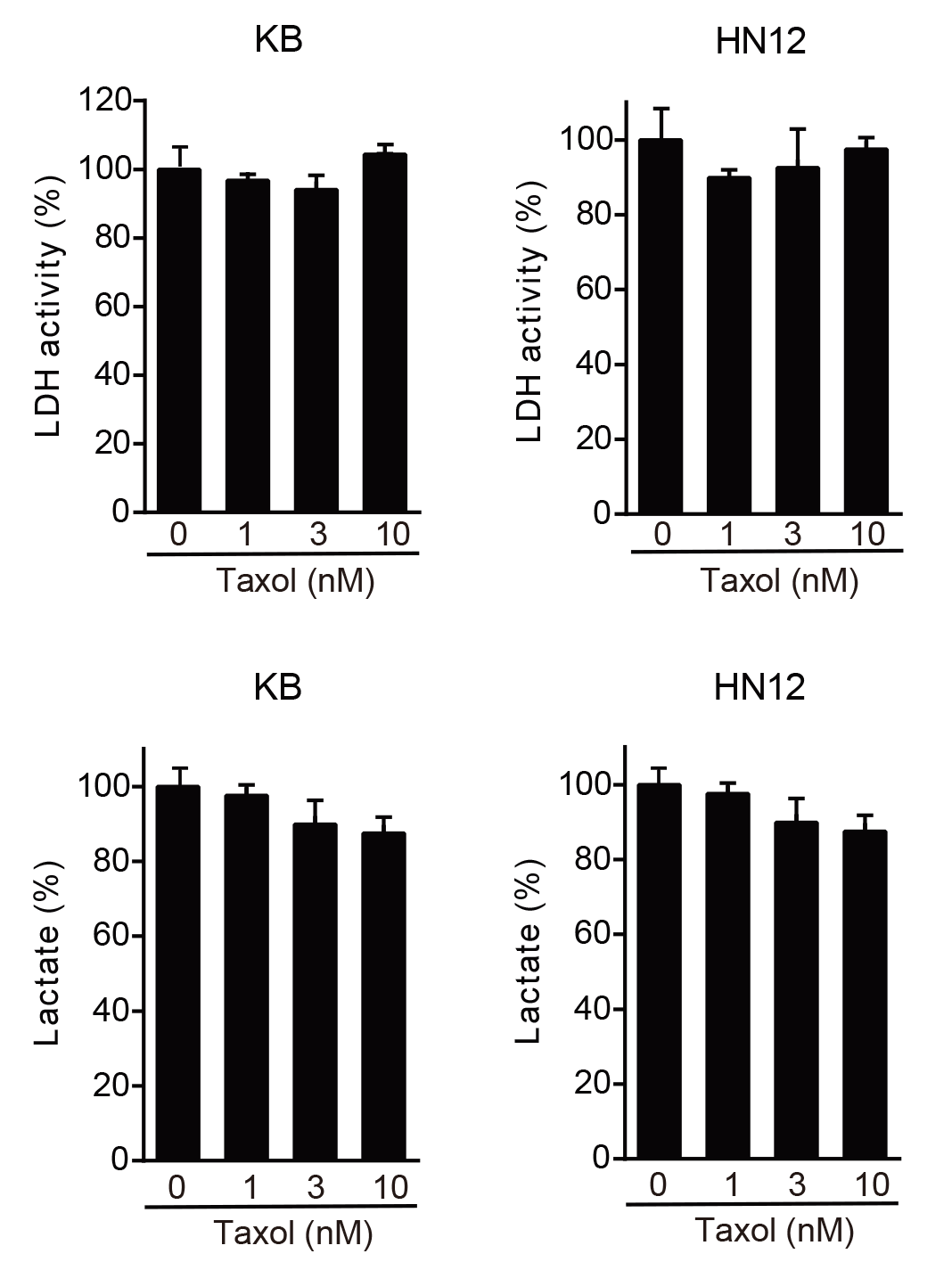

Supplement: S3 Fig — KB and HN12 cells were treated with taxol at the indicated concentrations. After 12 h, LDH activity and lactate amount were measured. Mean ± SE (n = 3). (TIF) [file pone.0125976.s003.tif]

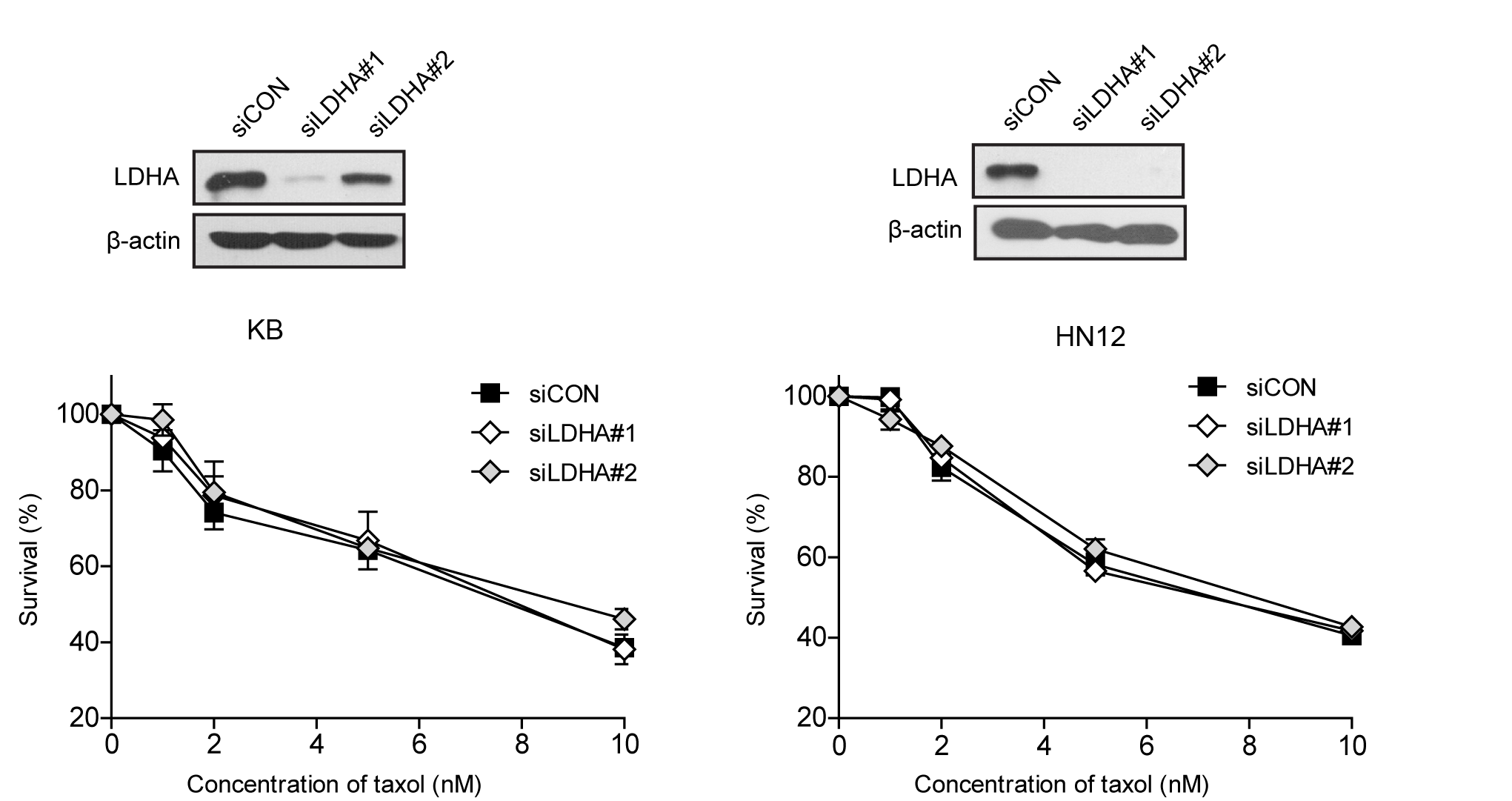

Supplement: S4 Fig — KB and HN12 cells were transfected with scrambled or LDHA siRNA and then treated with DMSO or taxol for 72 h at the indicated concentrations. Cell survival was examined by CCK8. Mean ± SE (n = 3). (TIF) [file pone.0125976.s004.tif]

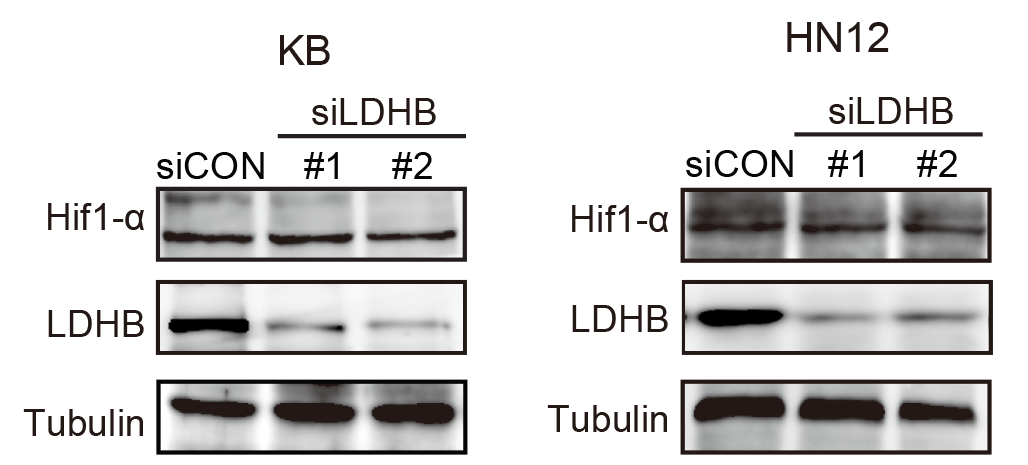

Supplement: S5 Fig — The efficiency of LDHB knockdown was verified by western blotting, followed by the detection of the expression of Hif-1α. (TIF) [file pone.0125976.s005.tif]

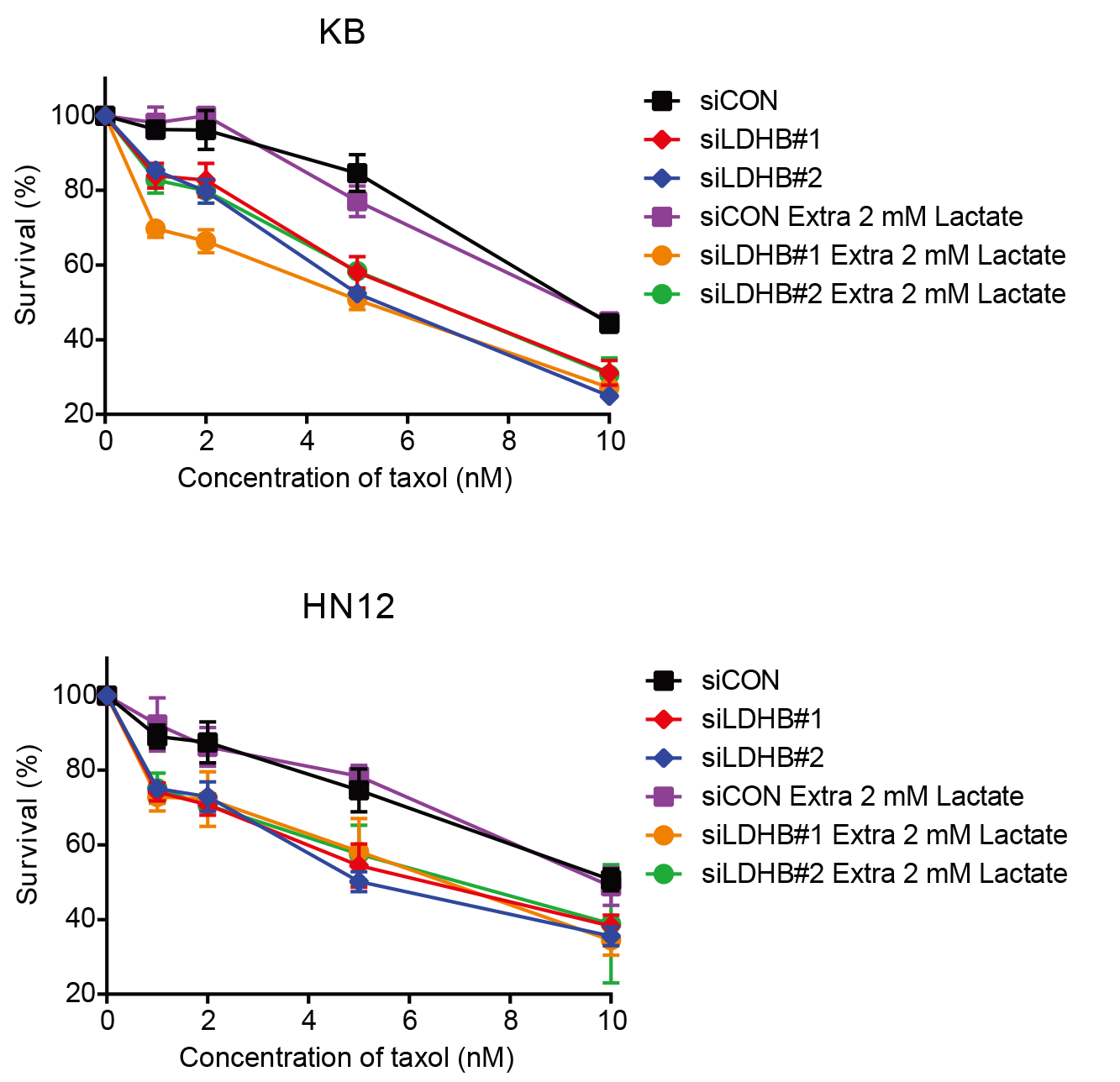

Supplement: S6 Fig — KB and HN12 cells were transfected with scrambled or LDHB siRNA and cell sensitivity was measured in the presence or absence of 2 mM lactate. Cell viability was measured using CCK8 assay following 72-h exposure to vehicle or taxol treatment. Mean ± SE (n = 3). (TIF) [file pone.0125976.s006.tif]

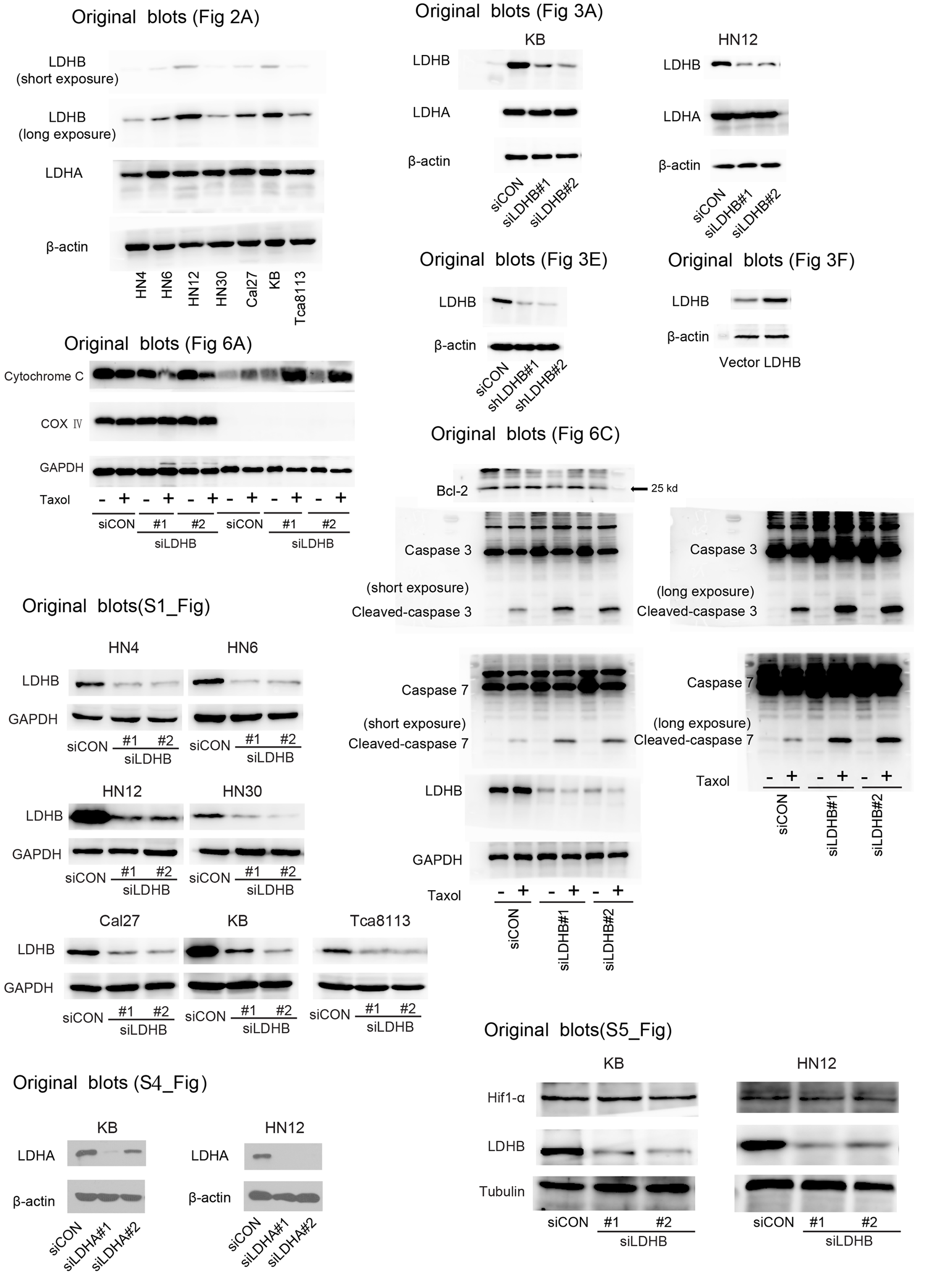

Supplement: S7 Fig — (TIF) [file pone.0125976.s007.tif]
